# Does Extended Intraoperative Peritoneal Lavage Really Bring Benefit on Patients With Gastric Cancer? A Meta-Analysis of Published Clinical Trials

**DOI:** 10.3389/fonc.2021.715040

**Published:** 2021-08-24

**Authors:** Wei Tao, Xiao-Yu Liu, Yu-Xi Cheng, Bing Kang, Hua Zhang, Chao Yuan, Bin Zhang, Dong Peng

**Affiliations:** ^1^Department of Gastrointestinal Surgery, The First Affiliated Hospital of Chongqing Medical University, Chongqing, China; ^2^Department of Clinical Nutrition, The First Affiliated Hospital of Chongqing Medical University, Chongqing, China

**Keywords:** extended intraoperative peritoneal lavage, gastric cancer, meta-analysis, outcomes, overall survival

## Abstract

**Purpose:**

The purpose of the current meta-analysis is to analyze whether extended intraoperative peritoneal lavage (EIPL) can bring benefit on short-term outcomes or survival for patients undergoing curative gastrectomy for gastric cancer.

**Methods:**

The PubMed, Embase, and Cochrane Library databases were searched from inception to May 3, 2021, to find eligible studies. Postoperative complications, overall survival (OS), disease-free survival (DFS), and peritoneal recurrence–free survival (PRFS) were compared between EIPL group and No EIPL group.

**Results:**

A total of five randomized controlled trials with 1,790 patients were included in the current meta-analysis. No difference was found in baseline information (p > 0.05). After pooling up the data of overall postoperative complications, no significant difference was found between EIPL group and No EIPL group (OR = 0.88, 95% CI = 0.51 to 1.53, P = 0.65). Furthermore, there was no significant difference between EIPL group and No EIPL group in terms of OS (HR = 0.77, 95% CI = 0.36 to 1.64, P = 0.49), DFS (HR = 0.97, 95% CI = 0.71 to 1.33, P = 0.87), and PRFS (HR = 1.03, 95% CI = 0.74 to 1.43, P = 0.86). In terms of subgroup analysis of OS, no significant difference was found as well (HR = 1.05, 95% CI = 0.82 to 1.34, P = 0.69).

**Conclusions:**

EIPL did not bring benefit in terms of short-term outcomes or survival. Therefore, EIPL is not recommended for patients undergoing curative gastrectomy for gastric cancer.

## Introduction

Gastric cancer is the fifth most common cancer in the world and the third leading cause of cancer-related deaths, especially in East Asia (1), and gastrectomy is still the main treatment for gastric cancer (2–4). Peritoneal metastasis (PM) is the main method of distant metastasis in gastric cancer and the main cause of cancer-related mortality. PM is detected in 10–30% of patients with gastric cancer at the time of initial diagnosis (5, 6), and furthermore, more than 50% of patients with stage II–III tumors develop PM within 5 years after gastrectomy (7, 8). Once PM occurs, symptoms such as refractory peritoneal effusion and cachexia may appear, which are the main causes of death (9).

The occurrence of PM may be caused by free cancer cells shed from the surface of the gastric serous membrane (10, 11). In addition, the operation of gastrectomy or lymph node dissection may cause cancer cells to fall off (12, 13). The elimination of free cancer cells during gastrectomy may effectively reduce the peritoneal recurrence of gastric cancer. Extensive intraoperative peritoneal lavage (EIPL) is a treatment method for preventing free cancer cells. The procedure of EIPL is as follows: the abdominal cavity is repeatedly flushed with 1 L of normal saline (up to 10 times) after gastrectomy (14, 15).

However, the complications and survival of EIPL treatment of gastric cancer are controversial. Previous studies have reported that EIPL could prolong overall survival and reduce complications (16, 17). However, other studies have shown that EIPL does not bring significant survival benefits and complications (18–20). Therefore, the purpose of the current meta-analysis is to analyze whether EIPL can bring benefit on short-term outcomes or survival.

## Methods

This meta-analysis was conducted in accordance with the Preferred Reporting Items for Systematic Reviews and Meta-Analyses (PRISMA) statement (21).

### Literature Search Strategy

The PubMed, Embase, and Cochrane Library databases were searched from inception to May 3, 2021, to find eligible studies. There were two key items, namely, extended intraoperative peritoneal lavage and gastric cancer. The search strategy for extended intraoperative peritoneal lavage was as follows: “extended intraoperative peritoneal lavage” OR “EIPL”. The search strategy for gastric cancer was as follows: “gastric cancer” OR “gastric carcinoma” OR “gastric neoplasms” OR “stomach cancer” OR “stomach carcinoma” OR “stomach neoplasms.” Then, we combined the two key search items using “AND”; moreover, the language was limited in English.

### Inclusion and Exclusion Criteria

The inclusion criteria were as follows: (1) patients who underwent EIPL and standard gastrectomy for gastric cancer; (2) the EIPL and No EIPL treatments were both reported; and (3) there was at least one outcome reported including postoperative complications and survival analysis. The exclusion criteria were as follows: (1) letters, comments, reviews, conferences, or case reports; and (2) insufficient data for extraction.

The inclusion and exclusion were conducted by two reviewers, respectively. Disagreement was settled by group discussion.

### Study Selection

The databases were searched by two reviewers independently. The titles and abstracts were screened after duplicate records were removed. After that, full texts were evaluated according to inclusion and exclusion criteria. Two reviewers conducted the study selection, and if disagreement occurred, final judgment was made by group discussion.

### Data Extraction

The data were extracted and cross-checked by two reviewers. The extracted data were as follows: the first author, publication year, country, study design, sample size, baseline information, surgical information, postoperative complications, and survival information.

### Outcomes and Definition

The primary outcome of the current meta-analysis was the survival analyses, which included overall survival (OS), disease-free survival (DFS), and peritoneal recurrence–free survival (PRFS). The secondary outcome was the postoperative complications, which included anastomotic leakage, pancreatic fistula, abdominal abscess, wound problems, postoperative bleeding, and short-term death.

The classification of postoperative complications was according to the Clavien-Dindo classification (22). OS was defined as the time from diagnosis to death from any cause. DFS was defined as the time from diagnosis to the time of recurrence, death, or last follow-up. PRFS was defined as the time from diagnosis to the time of peritoneal recurrence, death, or last follow-up.

### Quality Assessment

The criteria described in the Cochrane Handbook for Systematic Reviews of Interventions were used for evaluating the risk of bias in the included studies (23). Two reviewers conducted the bias evaluation, respectively.

### Statistical Analysis

In the current meta-analysis, continuous variables are presented as the mean and standard deviation (SD), and categorical variables are presented as proportions. For dichotomous and continuous variables, odds ratios (ORs) and mean differences (MDs) were calculated, and 95% confidence intervals (CIs) were calculated. The pooled hazard ratios (HRs) and the 95% confidence intervals (CIs) of each study were calculated to estimate the survival outcome. The I^2^ value and the results of the chi-squared test were used to assess the statistical heterogeneity (24, 25). High heterogeneity was considered when I^2^ > 50%; in such cases, the random effects model was used, and p < 0.1 was considered statistically significant. The fixed effects model was used when I^2^ ≤ 50%, and p < 0.05 was considered statistically significant. This meta-analysis was performed with RevMan 5.3 (The Cochrane Collaboration, London, United Kingdom).

## Results

### Study Selection

**Figure 1:**
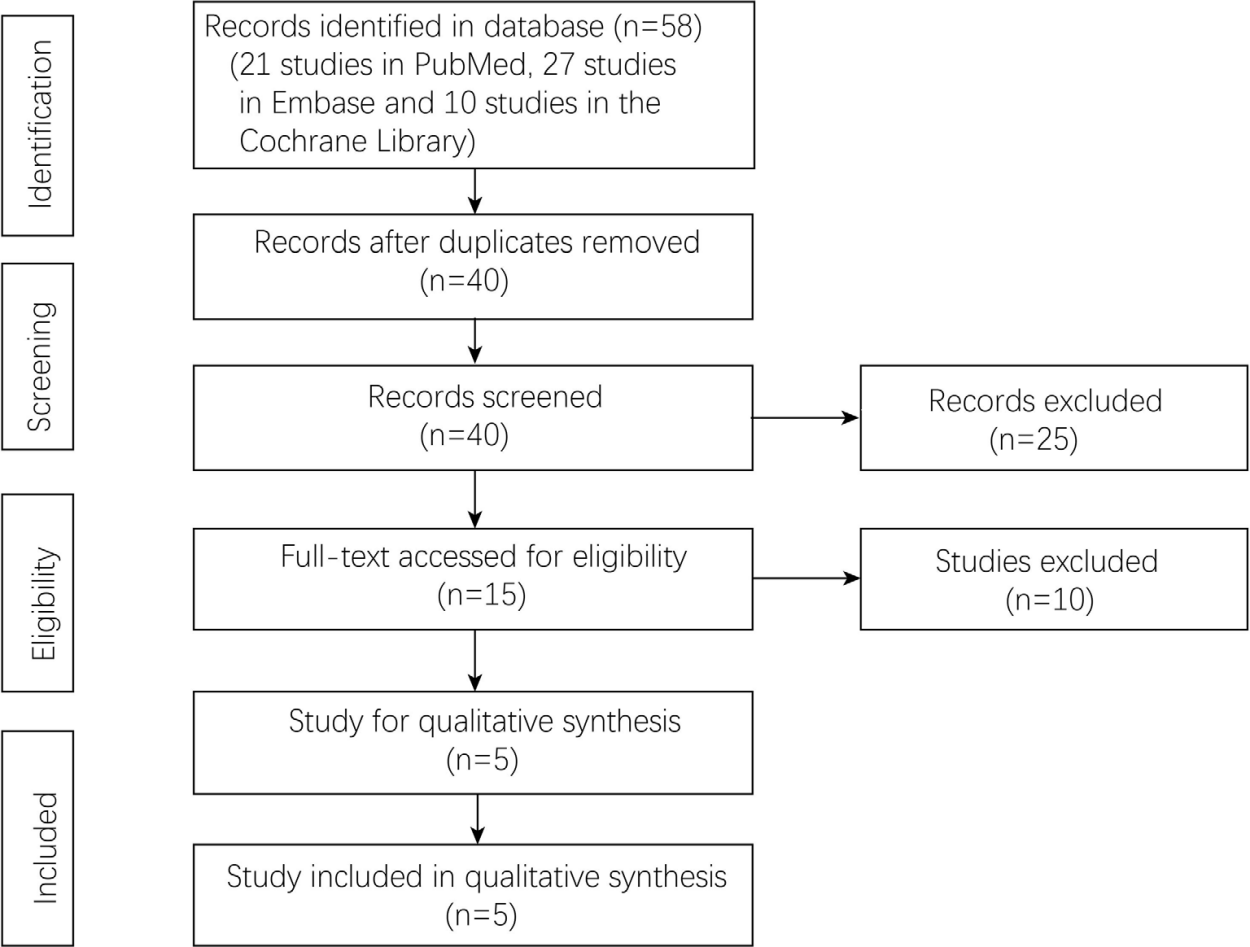
Flowchart of study selection.

A total of 58 studies (21 studies in PubMed, 27 studies in Embase, and 10 studies in the Cochrane library) were identified in the databases. Forty studies were left after removing the duplicates. Fifteen studies were accessed by full-text scanning, and finally, five randomized controlled trials (RCTs) were included (**Figure 1**).

### Characteristics of the Included Studies and Risk Bias Assessment

**Figure 2:**
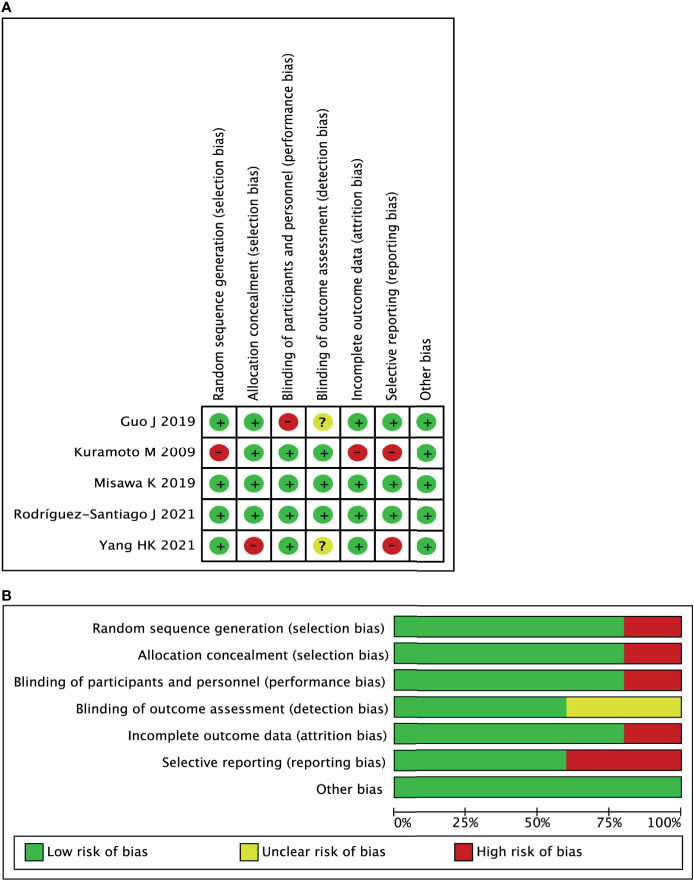
Risk of bias for each included study. **(A)** risk of bias summary. **(B)** Risk of bias graph.

A total of five studies (16–20) with 1,790 patients were included in the current meta-analysis. The publication year was from 2009 to 2021. Four RCTs were from Asia, and one RCT was from Spain. The detailed information about sample size and intervention method is shown in **Table 1**. Risk bias was accessed through seven aspects of the study, namely, random sequence generation, allocation concealment, blinding of the participants and personnel, selective reporting, incomplete outcome data, blinding of the outcome assessment, and other biases. The risk of bias summary and risk of bias graph are shown in **Figure 2**.

**Table 1:** Characteristics of the included studies.

Author	Year	Country	Study design	Method		Sample size		
				EIPL group	No EIPL group	Intervention	Control	Total
Misawa K	2019	Japan	RCT	Surgery+ EIPL	Surgery	145	150	295
Guo J	2019	China	RCT	Surgery+ EIPL	Surgery	279	271	550
Rodríguez-Santiago J	2021	Spain	RCT	Surgery+ EIPL	Surgery	43	43	86
Kuramoto M	2009	Japan	RCT	Surgery+ EIPL+ IPC	Surgery	30	29	59
Yang HK	2021	Singapore, Malaysia, Korea, China, and Japan	RCT	Surgery+ EIPL	Surgery	398	402	800

EIPL, extended intraoperative peritoneal lavage; RCT, randomized controlled trials; IPC, intraperitoneal chemotherapy.

### Summary of Information Between EIPL Group and No EIPL Group

The baseline information including age, sex, body mass index (BMI), American Society of Anesthesiologists (ASA), T and N stage were compared between EIPL group and No EIPL group, and no significant difference was found (p > 0.05). There was no significant difference in terms of surgical information including gastrectomy method, reconstruction method, and combined organ resection (p > 0.05) (**Table 2**).

**Table 2:** Summary of information between EIPL group and No EIPL group.

Characteristics	Studies	Participants (EIPL / No EIPL)	Mean Difference / Odds Ratio (95% CI)	Heterogeneity
Baseline information				
Age, year	4	497/ 493	−0.03 [−1.29, 1.23]; P=0.97	I^2^=0%; P=0.79
Male	5	895/ 895	0.98 [0.80, 1.20]; P=0.88	I^2^=0%; P=0.67
BMI, kg/m^2^	2	424/421	0.13 [-0.30, 0.56]; P=0.56	I^2^=0%; P=0.66
ASA 1–2	2	440/444	1.14 [0.72, 1.80]; P=0.58	I^2^=28%; P=0.24
ASA 3–4	2	440/444	0.88 [0.56,1.39]; P=0.58	I^2^=28%; P=0.24
T1–T3	4	862/864	0.95 [0.78, 1.16]; P=0.61	I^2^=0%; P=0.99
T4	4	862/864	1.05 [0.86, 1.28]; P=0.61	I^2^=0%; P=0.99
N0	4	865/866	0.94 [0.76, 1.18]; P=0.61	I^2^=0%; P=0.88
N1–N3	4	865/866	1.06 [0.85, 1.32]; P=0.61	I^2^=0%; P=0.88
Surgical information				
Total gastrectomy	5	895/895	1.07 [0.88, 1.29]; P=0.50	I^2^=29%; P=0.23
Roux-en-Y	2	441/445	1.12 [0.85, 1.48]; P=0.42	I^2^=0%; P=0.85
Combined organ resection	3	467/464	1.02 [0.69, 1.50]; P=0.92	I^2^=36%; P=0.21
Postoperative complications				
Anastomotic leakage	4	813/826	1.33 [0.65, 2.71]; P=0.43	I^2^=0%; P=0.79
Pancreatic fistula	4	813/826	0.51 [0.23, 1.13]; P=0.10	I^2^=0%; P=0.83
Abdominal abscess	4	813/826	0.87 [0.45, 1.66]; P=0.67	I^2^=29%; P=0.24
Wound problems	2	625/633	1.51 [0.61, 3.69]; P=0.37	I^2^=75%; P=0.05
Postoperative bleeding	2	625/633	0.70 [0.30, 1.64]; P=0.41	I^2^=7%; P=0.30
Short-term death	2	424/421	0.12 [0.02, 0.98]; P=0.05	I^2^=0%; P=0.69

EIPL, extended intraoperative peritoneal lavage; BMI, body mass index; ASA, American Society of Anesthesiologists; T, Tumor depth; N, lymph nodes.

Postoperative complications (Grade ≥ III) were graded by the Clavien-Dindo classification.

### Comparison of Complications Between EIPL Group and No EIPL Group

**Figure 3:**
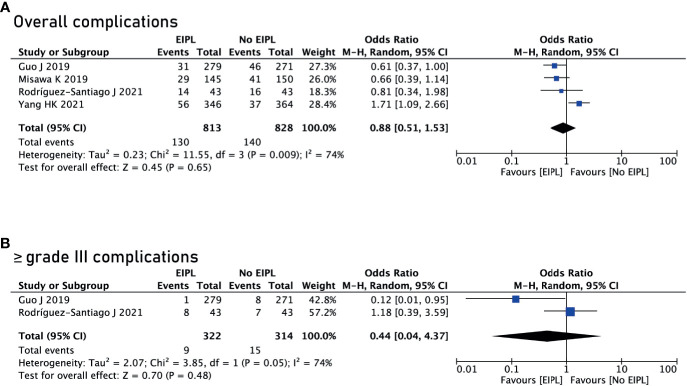
Comparison of complications between EIPL group and No EIPL group. **(A)** Overall complications. **(B)** ≥ grade III complications. EIPL, extended intraoperative peritoneal lavage.

Data regarding overall postoperative complications were extracted from four studies (16, 18–20). After pooling up the data, no significant difference was found between EIPL group and No EIPL group (OR = 0.88, 95% CI = 0.51 to 1.53, P = 0.65) (**Figure 3A**). In the subgroup meta-analysis of ≥ grade III complications, there was no significant difference between EIPL group and No EIPL group (OR = 0.44, 95% CI = 0.04 to 4.37, P = 0.48) (**Figure 3B**). The detailed postoperative complications including anastomotic leakage, pancreatic fistula, abdominal abscess, wound problems, postoperative bleeding, and short-term death were pooled up, and no significant difference was found between EIPL group and No EIPL group (p > 0.05).

### Survival Analysis Between EIPL Group and No EIPL Group

**Figure 4:**
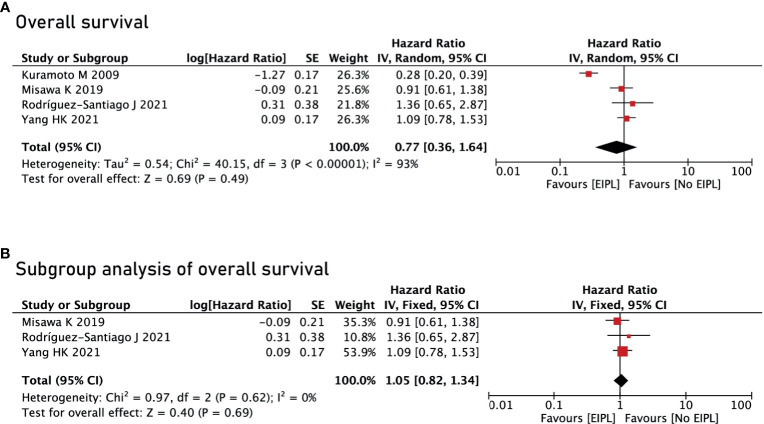
Overall survival analysis between EIPL group and No EIPL group. **(A)** Overall survival. **(B)** Subgroup analysis of overall survival. EIPL, extended intraoperative peritoneal lavage.

**Figure 5:**
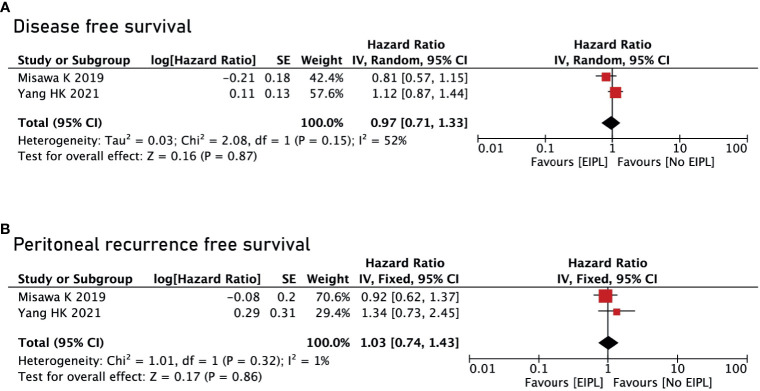
Disease-free survival and peritoneal recurrence–free survival between EIPL group and No EIPL group. **(A)** Disease-free survival. **(B)** Peritoneal recurrence–free survival. EIPL, extended intraoperative peritoneal lavage.

There were four studies reporting OS (17–20) and two studies reporting DFS and PRFS (18, 19). After pooling up all the data, no significant difference was found between EIPL group and No EIPL group in terms of OS (HR = 0.77, 95% CI = 0.36 to 1.64, P = 0.49) (**Figure 4A**), DFS (HR = 0.97, 95% CI = 0.71 to 1.33, P = 0.87) (**Figure 5A**), and PRFS (HR = 1.03, 95% CI = 0.74 to 1.43, P = 0.86) (**Figure 5B**). One of the five RCTs included intraperitoneal chemotherapy (IPC) (17); therefore, we did a subgroup analysis of OS between EIPL group and No EIPL group. Furthermore, after pooling the data, the heterogeneity was decreasing obviously, and no difference was found between EIPL group and No EIPL group (HR = 1.05, 95% CI = 0.82 to 1.34, P = 0.69) (**Figure 4B**).

## Discussion

A total of five RCTs with 1,790 patients were included in the current meta-analysis. No difference was found in baseline information. After pooling up the data of overall postoperative complications, no significant difference was found between EIPL group and No EIPL group. Furthermore, there was no significant difference between EIPL group and No EIPL group in terms of OS, DFS, and PRFS.

EIPL and IPC are immediate treatment during surgery. Some studies reported that IPC was effective and that IPC could bring some benefits (26, 27). However, others considered that IPC had a negative effect (17). Furthermore, hyperthermic intra-operative peritoneal chemotherapy (HIPEC) was thought to be another treatment for improving OS and DFS after gastrectomy (28). EIPL is also a currently controversial method. Previous studies reported that EIPL could prolong OS and reduce complications (16, 17). However, other studies have shown that EIPL does not bring significant survival benefits and complications (18–20). Therefore, it is necessary to analyze the exact efficacy of EIPL.

Guo J et al. (16) reported that EIPL could reduce the incidence of postoperative complications including intra-abdominal abscesses, surgical infection, and postoperative death. The probable mechanism was that EIPL was performed 10 times with 1 L of saline, and the dilution method could greatly reduce the amount of damaged tissues and wound exudates in the peritoneum and clean the peritoneal cavity. However, another study found that the EIPL group had higher adverse events (19). It might be related to bowel manipulation during lavage and higher number of superficial wound infections in the EIPL group. In this meta-analysis, we found that there was no difference in postoperative complications between the EIPL and No EIPL groups, and furthermore, there was no difference in severe complications. Therefore, EIPL might potentially clean the abdominal cavity, but it did not reduce the incidence of complications.

A large amount of saline was used immediately during the operation, which could theoretically reduce the metastasis of free cancer cells in the peritoneum and abdominal cavity (12, 13). Kuramoto M et al. (17) reported that the EIPL group improved OS compared to the No EIPL group. However, this study only included a small sample of 80 patients, and furthermore, the intervention group included IPC; therefore, the benefit of EIPL as a stand-alone therapy was still unknown. In this meta-analysis, after pooling up all the survival data, we found that there was no difference in terms of OS, and there was no difference between RFS or PRFS as well. It could be seen that EIPL had not achieved the expected results. However, there were only two research reporting RFS and PRFS, and the results might not be robust.

In addition to the overall postoperative complications and survival analysis, some studies reported some interesting clinical findings. Guo J et al. (16) reported that EIPL can significantly reduce postoperative pain, which might also be accidental. The probable reason was that the cause of pain reduction might be related to the reduction of inflammatory response. Pain is usually triggered by the local release of cytokines from inflammatory cells, which is a clinical reflection of tissue damage and inflammatory reactions (16, 29). Therefore, although EIPL was an interesting and simple intraoperative procedure, the current evidence could not support its widespread clinical application.

There were some certain limitations in the current meta-analysis. First, only five studies were included, which was relatively small; therefore, the results were not robust, and larger studies are needed. Second, DFS was chosen to be the primary outcome of the meta-analysis; however, peritoneal recurrence was difficult to detect from the included studies. Third, four RCTS were from Asian countries and one RCT was from western countries; the results might apply to Asian areas, and multicenter, multiregional high-quality RCTs should be carried out in the future.

In conclusion, EIPL did not bring benefit in terms of short-term outcomes or survival. Therefore, EIPL is not recommended for patients undergoing curative gastrectomy for gastric cancer.

## Data Availability Statement

The original contributions presented in the study are included in the article/supplementary material. Further inquiries can be directed to the corresponding author.

## Author Contributions

DP and WT contributed to conception and design of the study. X-YL organized the database. DP performed the statistical analysis. DP and WT wrote the first draft of the manuscript. Y-XC, HZ, CY, BZ, and BK wrote sections of the manuscript. All authors contributed to article and approved the submitted version.

## Conflict of Interest

The authors declare that the research was conducted in the absence of any commercial or financial relationships that could be construed as a potential conflict of interest.

## Publisher’s Note

All claims expressed in this article are solely those of the authors and do not necessarily represent those of their affiliated organizations, or those of the publisher, the editors and the reviewers. Any product that may be evaluated in this article, or claim that may be made by its manufacturer, is not guaranteed or endorsed by the publisher.
